# Functional principal component analysis for identifying the child growth pattern using longitudinal birth cohort data

**DOI:** 10.1186/s12874-022-01566-0

**Published:** 2022-03-21

**Authors:** Reka Karuppusami, Belavendra Antonisamy, Prasanna S. Premkumar

**Affiliations:** Department of Biostatistics, Christian Medical College, Vellore, 632002 India

**Keywords:** Cohort, Child growth, Functional principal component analysis, Longitudinal, Urban slums

## Abstract

**Background:**

Longitudinal studies are important to understand patterns of growth in children and limited in India. It is important to identify an approach for characterising growth trajectories to distinguish between children who have healthy growth and those growth is poor. Many statistical approaches are available to assess the longitudinal growth data and which are difficult to recognize the pattern. In this research study, we employed functional principal component analysis (FPCA) as a statistical method to find the pattern of growth data. The purpose of this study is to describe the longitudinal child growth trajectory pattern under 3 years of age using functional principal component method.

**Methods:**

Children born between March 2002 and August 2003 (*n* = 290) were followed until their third birthday in three neighbouring slums in Vellore, South India. Field workers visited homes to collect details of morbidity twice a week. Height and weight were measured monthly from 1 month of age in a study-run clinic. Longitudinal child growth trajectory pattern were extracted using Functional Principal Component analysis using B-spline basis functions with smoothing parameters. Functional linear model was used to assess the factors association with the growth functions.

**Results:**

We have obtained four FPCs explained by 86.5, 3.9, 3.1 and 2.2% of the variation respectively for the height functions. For height, 38% of the children’s had poor growth trajectories. Similarly, three FPCs explained 76.2, 8.8, and 4.7% respectively for the weight functions and 44% of the children’s had poor growth in their weight trajectories. Results show that gender, socio-economic status, parent’s education, breast feeding, and gravida are associated and, influence the growth pattern in children.

**Conclusions:**

The FPC approach deals with subjects’ dynamics of growth and not with specific values at given times. FPC could be a better alternate approach for both dimension reduction and pattern detection. FPC may be used to offer greater insight for classification.

**Supplementary Information:**

The online version contains supplementary material available at 10.1186/s12874-022-01566-0.

## Background

In developing countries, poor growth of children under five is a major public health problem. The study of physical growth in children is challenging and depends on many factors such as genetic, malnutrition, physiological and socio-economic factors [1–4]. Normal growth is the greatest indicator of children’s well-being and provides an accurate marker of inequalities in human development. This is reflected in the millions of children worldwide who fail to achieve their normal growth potential because of health conditions and inadequate care and nutrition. Children with poor growth has permanent impact on their physical and cognitive development [5]. The difficulty in visually identifying poor growth children and the lack of routine assessment of normal growth in primary health care services explain why it has taken so long to identify the magnitude of this hidden scourge. High amount of malnutrition experienced by children living in urban slum dwellers and similar settings may harmfully impact their health development of physical characteristics such as height or weight [6–8].

The new approach of functional principal component analysis (FPCA) has been used as a statistical method for analysing and characterising growth trajectory data [9]. The FPCA approach is suitable for extracting the pattern of entire growth as a function that would otherwise be lost when applying the traditional statistical techniques. Recently, the functional data analysis (FDA) is a statistical approach to handle the huge data and to detect the associations between one or more factors and a longitudinal growth outcome data [9, 10], but there is always a concern about the type of basis functions of the FDA framework. The challenges with functional data approach lie in the assumption of smoothness, variability in the time direction and alignment of the functions. The well suitable basis system should also be explored.

A B spline basis is used for flexibility of the data [11]. A spline based smoothing is especially useful for fairly smooth and closely monotonic structure of the functions or trajectories. It is allowing to extract the features from growth data [11, 12]. The FPCA is one of the popular analysis techniques under FDA and used to extract the information from functional data. This approach is used as dimension reduction in functional data and successfully applied to real life scenarios analysis such as study of cornea in the human eye [13], fMRI scans in the human brain [12, 14], foetal movement monitoring data [15], gene expression profiles [16] and growth study [9, 10]. Many more various applications of FPCA have been developed.

The more flexible FPCA could be used to find temporal variations in growth data. Other interesting feature of FDA is to study the relation between longitudinal outcome and factors. Such models are named functional linear models (FLMs). The primary aim of this study is to characterize individual growth trajectories of children in the first 3 years of life using Functional Principal Component (FPC) analysis under well-established FDA framework.

## Methods

### Birth cohort study

The design of the study has been reported earlier [17–19]. Longitudinal birth cohort study was conducted in three neighbouring urban slums in Vellore measuring 2.2 sq.km with a population density of approximately 17,000 per sq.km, South India. The data were collected from these three slums Kaspa, Ramnaickanpalayam and Chinnallapuram where the living environment is poor such as open drains, without water and toilets, without secure tenancy, overcrowded clustered houses with many rubbish dumps. The common occupation in the study area is the manual production of tobacco based beedi products for a daily wage.

**Fig. 1:**
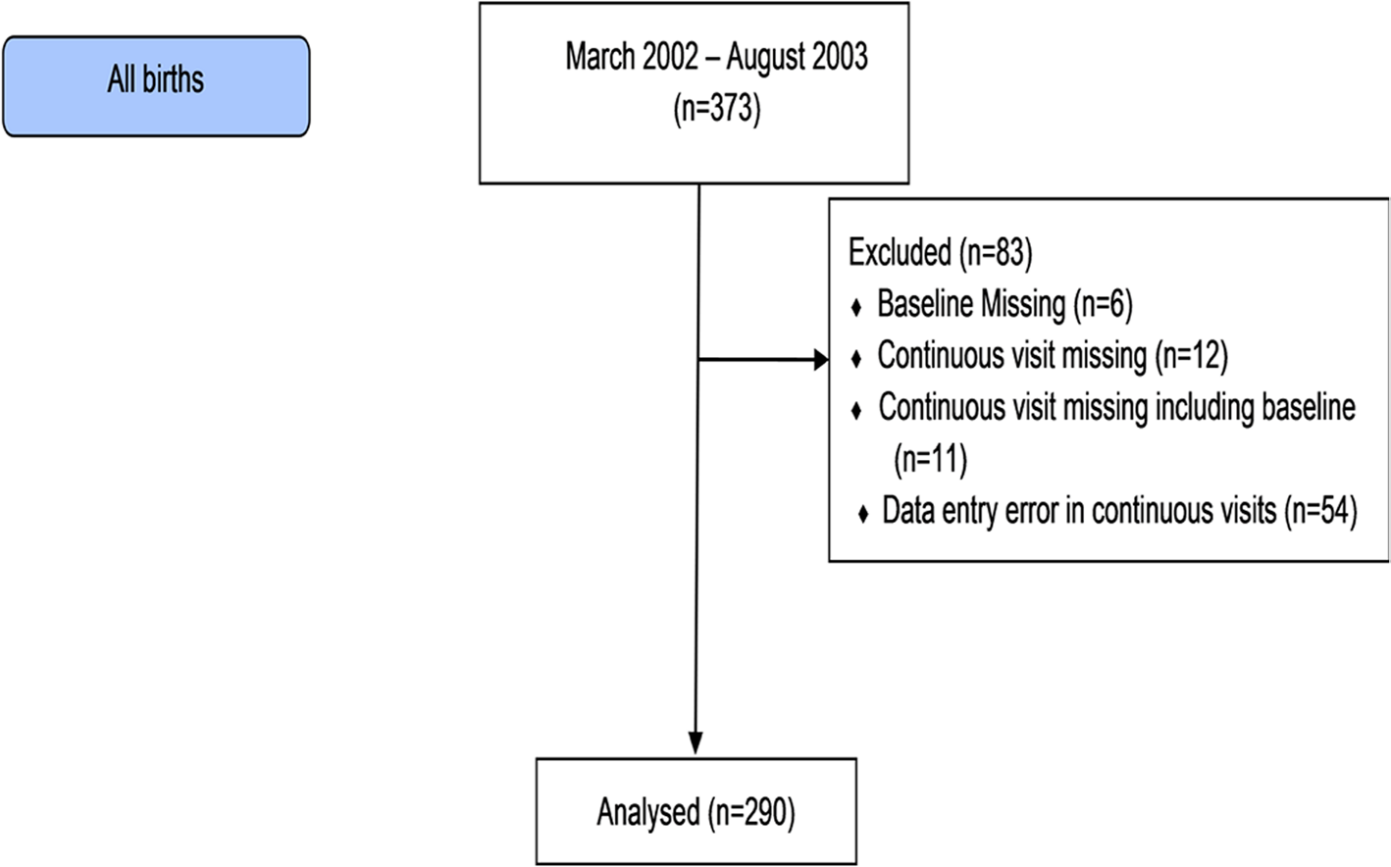
Flow chart of study participants

Women of child-bearing age were visited to identify new pregnancies during a survey conducted in 2002. Children of pregnant women intending to remain in the area for 3 years were eligible for enrolment. Infants were recruited from birth between March 2002 and August 2003 following written informed consent from the mother. These children were followed until their third birthday. The last child was followed up to August 2006. This study was approved by the Institutional Review Board and ethics committee of Christian Medical College and Hospital. In this study, 290 children were included (Fig. 1). In the original study, children were visited twice a week to record incidence of diarrhoea and morbidities. Weight and length at birth were obtained from delivery records available at the first home visit. Subsequently, height and weight were measured at every month until 36 months by field workers at the study clinic using single measurements. Recumbent length was measured using a standard infantometer and subsequently using a stadiometer, both to the nearest millimetre. Weight was measured using a Salter weighing scale to the nearest 100 g. Due to missing growth measurements beyond 3 years of follow up, we have included height and weight from birth to 36 months for the study data analysis.

### Study variables

Baseline study characteristics of interest included were gender, height (cm), weight (kg), baby in ICU or not, abortion (yes, no), mode of delivery (suction, forceps, caesarean, vaginal), socio economic status (low, middle, high), gravida (1,2,3, > 3), highest education of household (no formal education, Primary school (1-5 years), Middle school (6-8 years), High school (9-10 years), Higher secondary / College/ Polytechnic / Professional (> 10 years)), and duration of exclusive breast feeding (< 3 months, ≥ 3 months).

### Statistical analysis

For demographic and other characteristics, data are presented as mean and standard deviation (SD) for normally distributed variables, and as frequency (percentage) for categorical variables. There were few values missing in the follow-up visits of the growth outcomes. Using the Last Observation Carry Forward (LOCF) method of imputation, the data was considered as complete dense data. To handle and analyse the large amount of constantly measured growth data, Functional Data Analysis (FDA) framework was used [11, 12, 20–24].

### Smoothing and B-spline basis functions

Assuming that a curve or function for replication ‘*i’* arrives as a set of measured values, *y*_*i*1_, *y*_*i*2_, …, *y*_*i*n_, the first step is to convert these values into a curve or function *x*_i_ with values *x*_i_(t) computable for argument value at time ‘t’. A set of functional building blocks *ɸ*_*k, k = 1,2,…,K*_ which is called basis functions and are combined linearly. A function or curve x(t) is expressed in mathematical representation as

1 $$x(t)=\sum_{k=1}^K{c}_k\ {\phi}_k(t)$$

in terms of large number K known basis functions *ϕ*_*k*._

Where c indicate the vector of length K of the coefficients *c*_*k*_ and *ϕ* as the functional vector whose elements are the basis functions *ϕ*_*k*_.

Spline functions are the common choice of approximation system for the functional data in the specific nature. It has more or less replaced polynomials, which in any case they contain within the system. In defining a spline, the first step is to divide the interval over which a function is to be approximated into *S* subintervals separated by values

$${T}_{s,}\ s=1,2,\dots, S-1$$

and which are called knots.

A spline function is a polynomial of specified order m in each interval. To construct the child growth outcome trajectories into functions, we have applied B-spline system. To construct the basis function, number of order, knots and range were chosen. Using these information along with the number of basis then the B spline basis was generated. A B splines-based smoother is used because its simplicity and flexibility for data [11, 12, 21–24].

### Outlying function

Outlier detection visualizing tools such as Functional version of Box plot and outliergram were used to identify an abnormal function in both outcomes [12, 24–26]. There are two types of variability in the functions: (i) amplitude variation and (ii) phase variation. The amplitude variation deals with the differences in height between the functions. The phase variation deals with the differences in timing of important features between the functions. The registration technique was carried out to improve the curve misalignment [12, 23, 27, 28].

### Functional principal component analysis

FDA is an advanced statistical methodology specially established for analysing temporal data [29]. The longitudinal child growth trajectories was converted into functions using the B-spline basis with smoothing parameter (λ) and which is chosen by the generalized cross-validation (GCV) technique [30]. An optimal of smoothing parameter for growth and other temporal data is generally recommended [31]. This smoothing approach eliminates the random noise from month wise data. Functional principal component analysis (FPCA) is an extension of conventional principal component analysis (PCA) to functional data [29]. We applied Functional version of PCA to identify the important temporal pattern across the growth smooth functions. Individual monthly growth observations *x*_*i*_ are replaced with smooth functions *x*_*i*_(*t*) in the functional setting [29] and weighting coefficient functions *β*_*j*_(*t*).

2 $${f}_i=\int \beta (t)\kern0.5em {x}_i(t)\kern0.5em d\kern0.5em t,\kern0.5em \mathrm{i}=1,2\dots, \mathrm{N}$$

The FPCA was used to extract the information from functional data to identify the different pattern of the child growth function. Independent functional principal component curves describe the important modes of temporal variability in growth across the individual fitted curves. FPCA also reduces the dimensions of the problem by representing functions in terms of a finite set of functions and further functional linear model was used to assess the association between factors and trajectories [20, 22–24, 32–36]. The conditional kernel density estimators plot was used to identify the subgroup of the growth functions and, the proportion of children contributing to each subgroup were estimated.

### Functional linear model

The traditional statistical methods of analysis of variance (ANOVA) and linear regression investigates the variability in observed data can be accounted for by other known variables. Functional version regression models are used for modelling relation between functional and non-functional variables. When the independent variable is categorical and the outcome is functional, our interest is to determine whether there are differences in the functional outcome among the different categories of the independent variable. In functional setting, the response variable *y* with argument t is functional version. The most general linear model is,

3 $${y}_i(t)={\beta}_0(t)\kern0.5em +\kern0.5em {\sum}_{j=1}^p{\beta}_i(t)\kern0.5em {x}_{ij}$$

Further this was applied and explored to growth data to assess the relation between variables and trajectories.

### Software

All statistical analysis were performed using R studio version 3.6.1. FDA was performed using fda package.

## Results

### Sociodemographic characteristics

The demographic characteristics of the study sample are detailed in the Table 1. In this study, male and female were almost equally distributed as 49.3 and 50.7%. Baseline mean height (cm) and weight (kg) of the children were 52.75 (SD: 3.28) and 3.65 (SD: 0.71) respectively and 281 children (96.9%) did not have ICU admissions. Formal education was not attained in 7.2% of the household and 62.4% were from low socio-economic status. About 14% mothers had more than one abortion and 17.9% of mothers had more than three pregnancies. Normal delivery was reported in 91.7% mothers and 52.7% of women exclusively breastfeed their children for less than 3 months.

**Table 1:** Sociodemographic characteristics

Characteristics	*n* = 290
	n(%)
Height (cm), mean (SD)	52.75 (3.28)
Weight (kg), mean (SD)	3.65 (0.71)
**Gender**	
Male	143 (49.31)
Female	147 (50.69)
**Baby in ICU**	
Yes	9 (3.10)
No	281 (96.90)
**Abortion**	
Yes	40 (13.79)
No	250 (86.21)
**Mode of delivery**	
Suction or Forceps or Ceasarean	24 (8.28)
Vaginal	266 (91.72)
**Socio Economic status**	
Low	181 (62.41)
Moderate/High	109 (37.59)
**Gravida**	
1	76 (26.21)
2	93 (32.07)
3	69 (23.79)
>3	52 (17.93)
**Highest Education of Household**	
Illiterate	21 (7.24)
Primary	71 (24.48)
Middle	83 (28.62)
High	79 (27.25)
Higher secondary/College/Polytechnic/Professional	36 (12.41)
**Duration of exclusive breast feeding**	
< 3 months	153 (52.76)
≥ 3 months	137 (47.24)

**Fig. 2:**
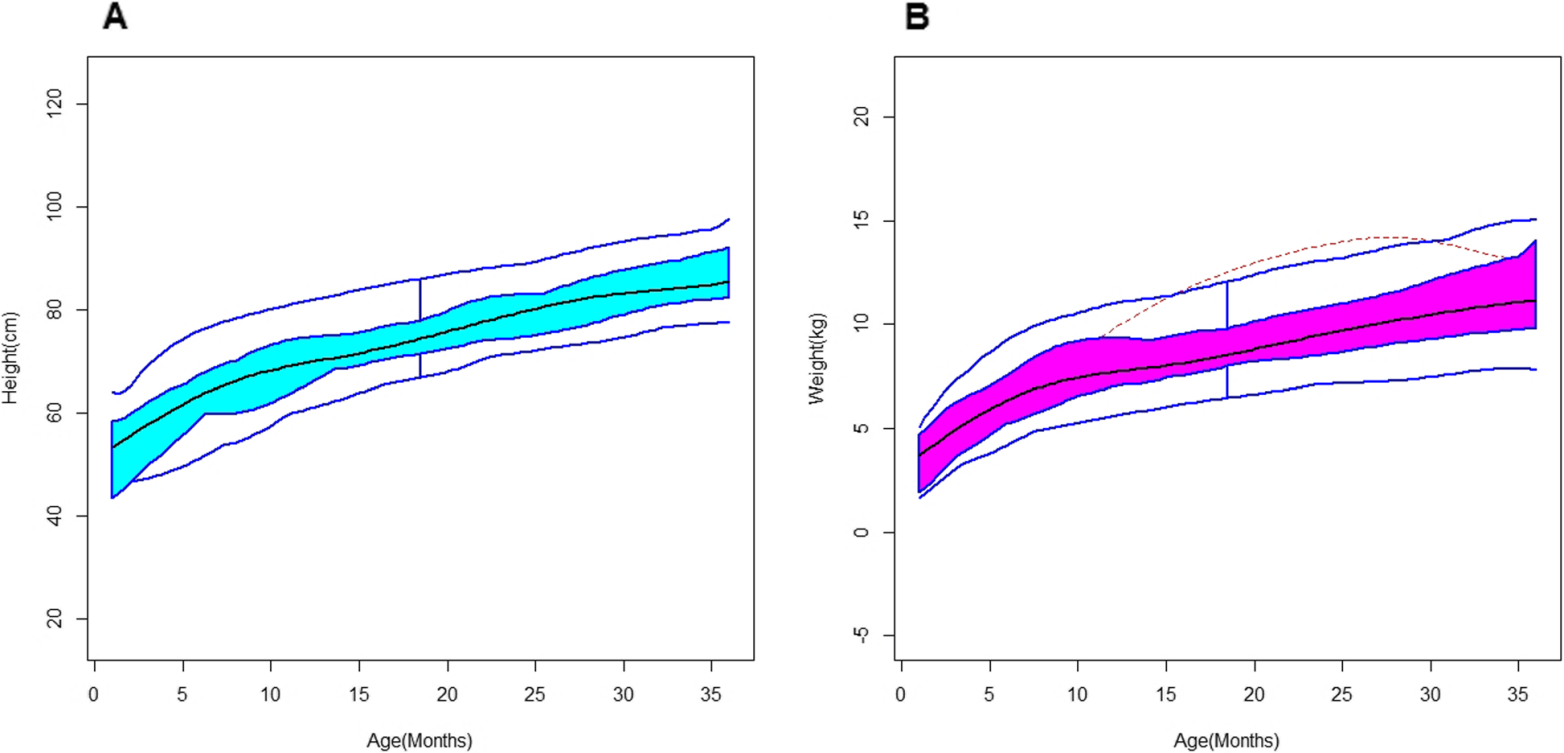
Functional boxplots of height and weight functions with a black curve representing the median curve, aqua green and pink area denoting the 50% central region, the two inside blue curves indicating the envelops of 50% central region, the two outside blue curves indicating for two non-outlying extreme curves, and the red dashed curve representing the outlier candidates. **A** Functional boxplots of Height function. **B** Functional boxplots of Weight function

**Fig. 3:**
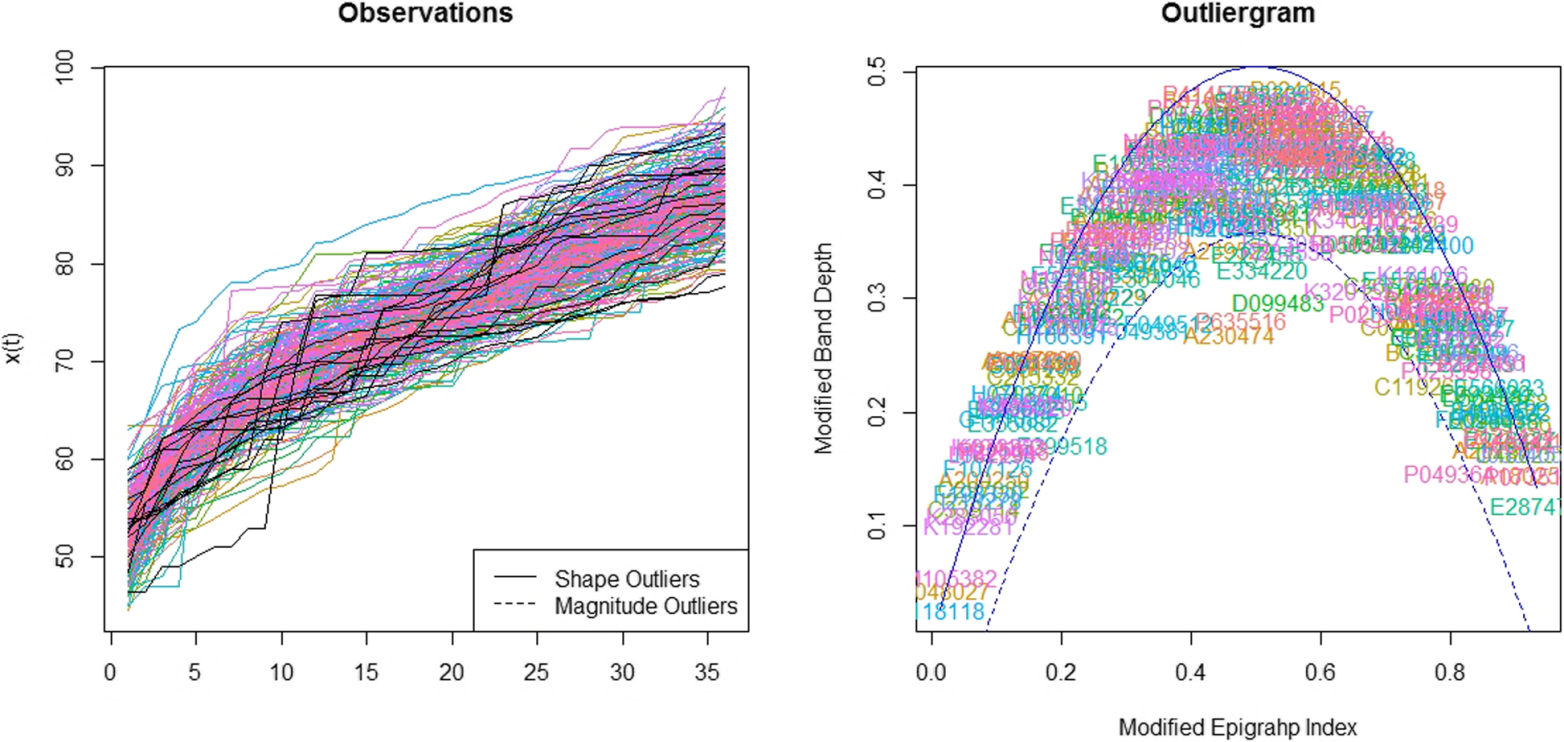
The outliergram plot for height functions. Right: Modified band depth versus modified epigraph index of the 290 functions. The solid parabola and the dashed one represents the boundary between outlying and non-outlying observations. Left: Height functions of 290 children during ages between 0 and 36 months

**Fig. 4:**
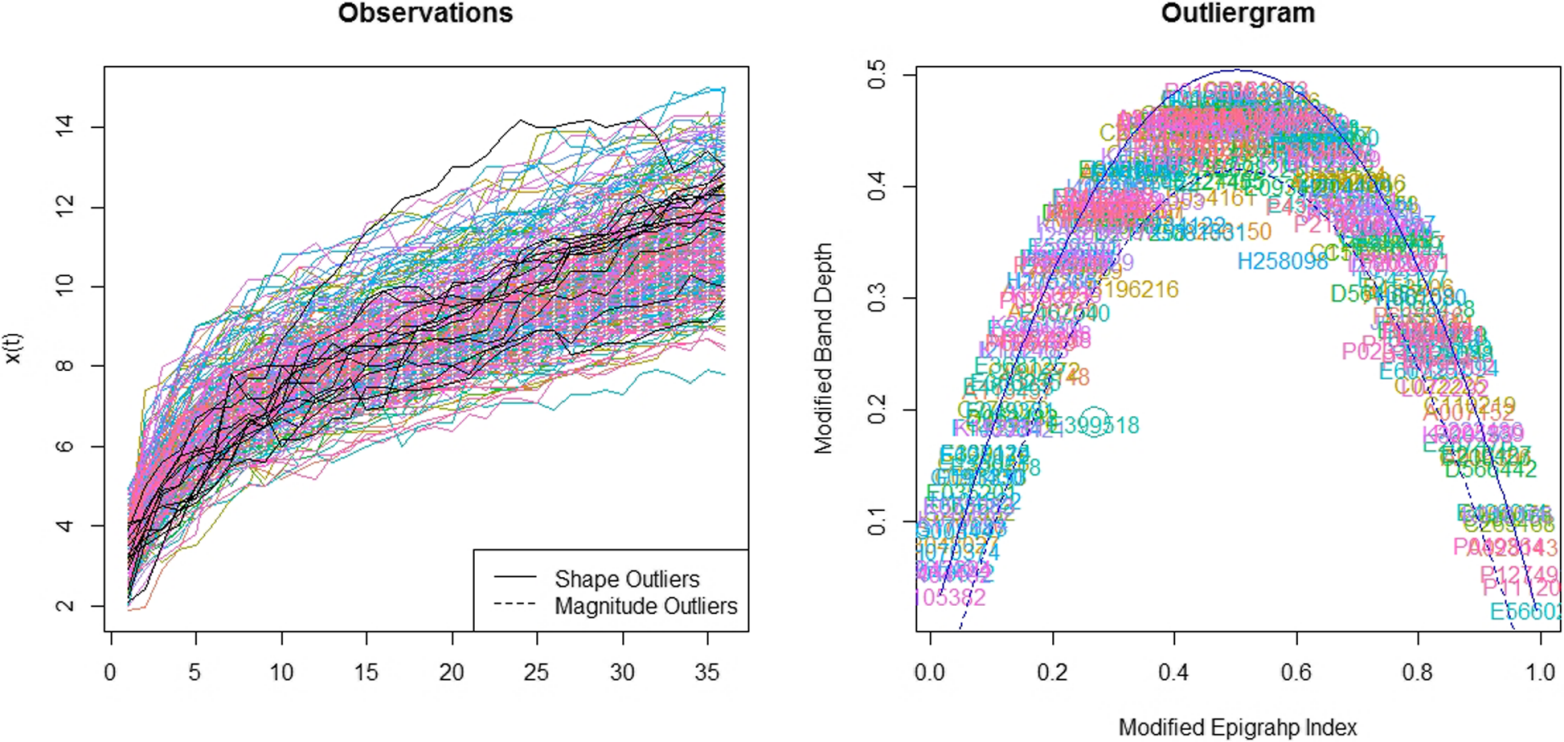
The outliergram plot for weight functions. Right: Modified band depth versus modified epigraph index of the 290 functions. The solid parabola and the dashed one represents the boundary between outlying and non-outlying observations. Circle stand for outlier (subject id). Left: Weight functions of 290 children during ages between 0 and 36 months

Considering the generated B spline basis, smoothing parameter and second order of penalization, the functions were generated for growth outcomes. The Functional Box plot is shown in Fig. 2. It was plotted as x axis will be taken as age (months) and y axis will be taken as growth parameter values. Besides the 50% central region, the 25 and 75% central regions were provided as well. It is important to note that the box, the whiskers, and the median can reveal useful information about a functional dataset by looking at their size, position, length, and even the shape of the box or the central tendency of median function. In Figs. 3 and 4, there are two measures named Modified Epigraph Index and Modified Band Depth, using these two in x and y axis the plots were generated as outliergram for height and weight functions. These two measures provide an idea of how central a function is with respect to a set of functions. A shape outlier function was noted and confirmed as abnormal function by these two methods and the abnormal function is excluded from the weight functions. There was no magnitude outlier in the functional data.

### Functional principal component analyses

**Fig. 5:**
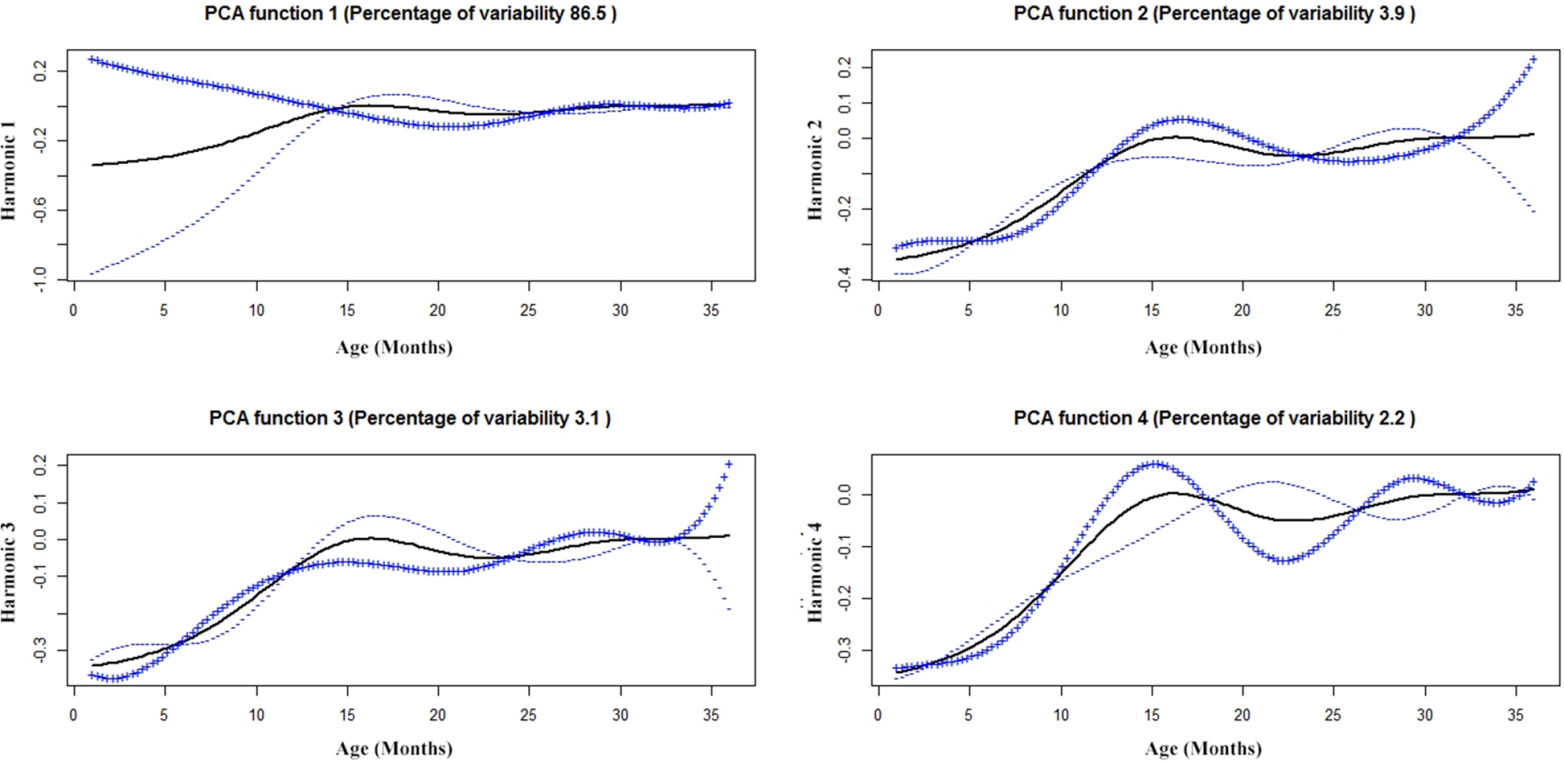
A principal component analysis of aligned 290 height trajectories. The first four important harmonics, each plot shows the mean function (solid black) +/− small amount of harmonics or functions obtained by adding or subtracting from mean function. The x-axis denotes the age in months

Child growth data contains two main time-varying traits; height, and weight. We obtained patterns of variation in the growth outcomes by using FPCA. We obtained the first four FPC related to the height functions and the plot shown in Fig. 5. The first, second, third and fourth FPCs explained 86.5, 3.9, 3.1 and 2.2% of the variation respectively. The first two FPC explained 90.4% of the variability and 95.7% of the variability was explained by the first 4 FPC. Except the first Eigen function, remaining all are less important. FPC of height function explains that, component 1 accounts for higher deviation from mean for first 12 months, component 2 and 3 are in contrast to component 1 because the deviation occurs after 30 months. Component 4 accounts less deviation before 10 months and after 32 months. These four subgroups correspond to different height patterns, which can be labelled as “poor growth”, “general or normal growth”, “catch up” and “growth acceleration”.

**Fig. 6:**
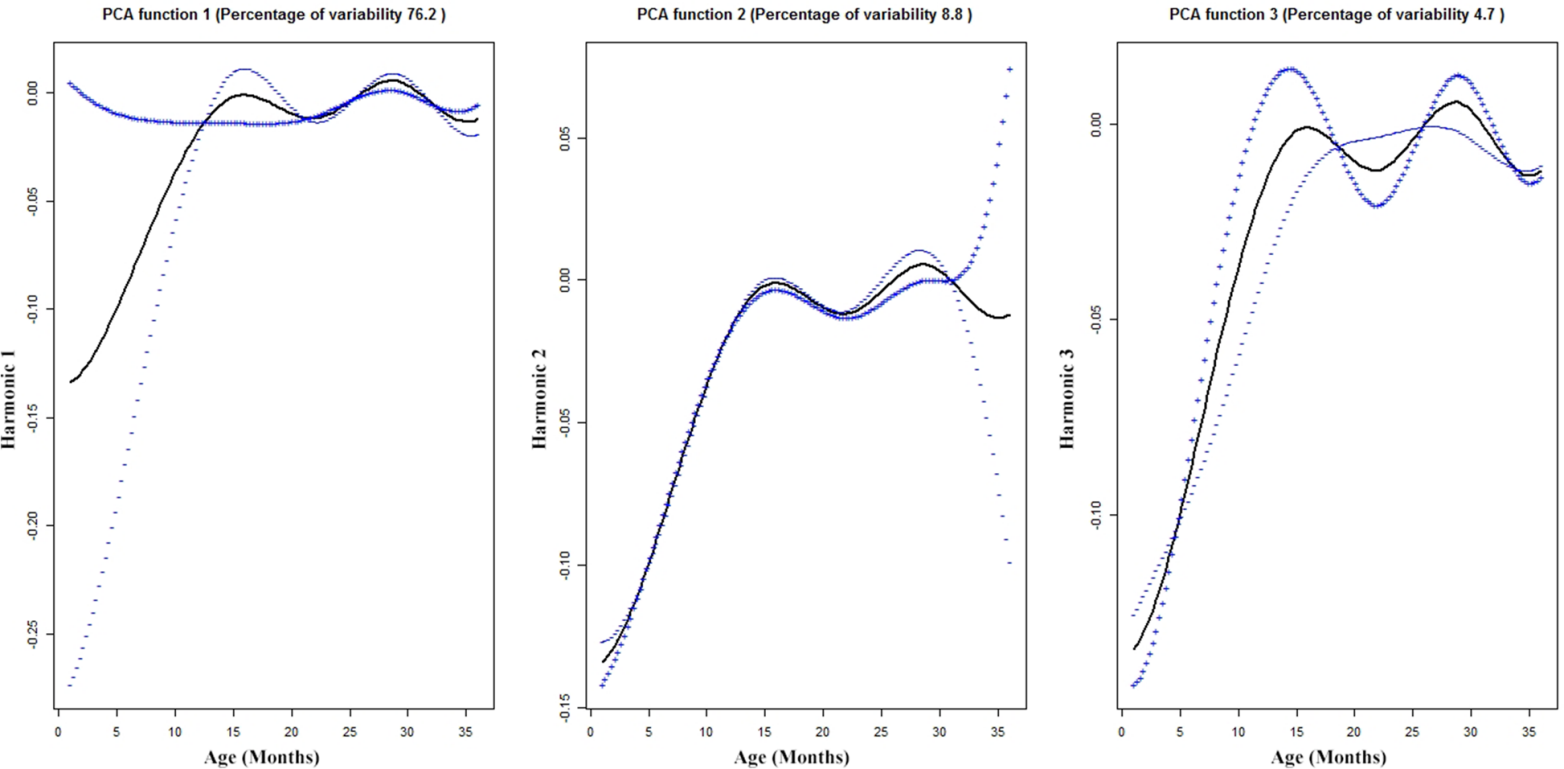
A principal component analysis of aligned 289 weight trajectories. The first three important harmonics, each plot shows the mean function (solid black) +/− small amount of harmonics or functions obtained by adding or subtracting from mean function. The x-axis denotes the age in months

**Fig. 7:**
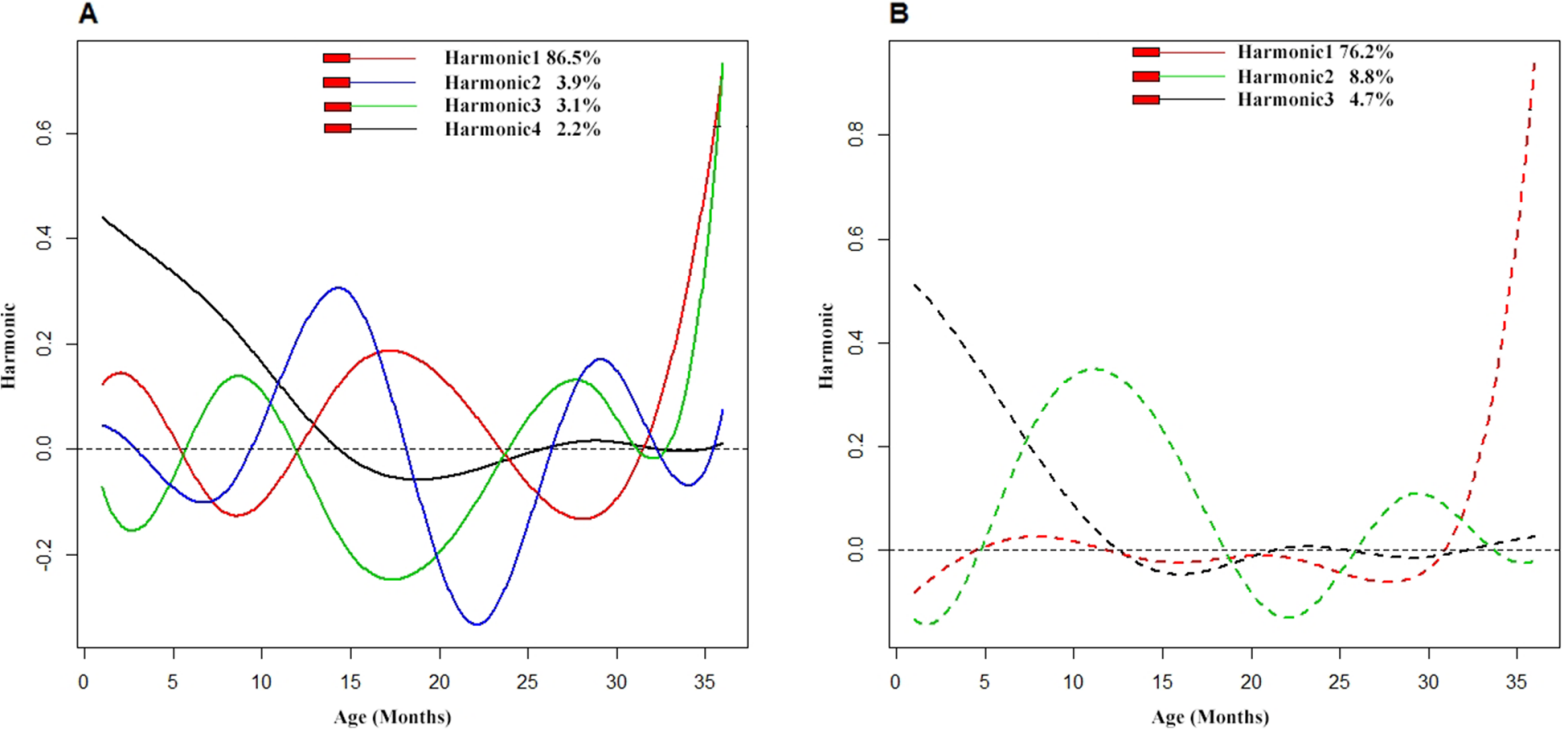
**A** Eigen plots for height functions (**B**) Eigen plots for weight functions

The first three FPC related to the weight functions are shown in Fig. 6. The first, second and third FPCs explained 76.2, 8.8 and 4.7% of the variation respectively. First two FPCs accounted totally 85% of the variability and 90% of the variability was explained by the first 3 FPCs. Component 1 accounts for higher deviation from mean for first 12 months, component 2 is in contrast to component 1 because the deviation occurs after 30 month and overall component 3 accounts less deviation from the mean. These three subgroups correspond to different weight pattern, which can be labelled as “poor growth”, “general or normal growth” and “growth acceleration“. The Eigen plots for height and weight functions are given in Fig. 7. About 38% (111/290) of the children had poor growth in height and 44% (128/289) of the children had poor growth in their weight function.

### Functional responses and an analysis of variance

To examine the factors affecting the growth functions, functional linear model was used to assess the association between growth function and factors such as gender, socio economic status, duration of breast feeding, gravida, and highest education of house hold. The regression coefficient plot with confidence interval are given in the Additional files Figs. 1, 2, 3, 4, 5, 6, 7, 8, 9, 10. The results show (i) Male children had growth increments on height and weight function during the first 10 months and 6 months respectively. (ii) Low socio-economic status had shown a poor growth after 6 months on height function and for weight after 3 months compared to children belonging to middle and high socio-economic status. (iii) Children not exclusively breast fed for more than 3 months displayed poor growth category on height and weight function. (iv) Children born to the parents who are illiterate and primary education had poor growth on height and weight function. (v) Children born to the women with higher order of gravida (≥ 3) had poor growth on height and weight function.

## Discussion

In public health and environmental research, repeated measures are occasionally obtained at rapid frequencies over longer period of time. In this scenario, using the conventional technique to analyse large amount of data will be difficult. Functional methods provide an alternative flexible approach to common parametric models for analysing panel data and are computationally efficient and easy to implement. This study outlines a modern statistical framework for handling the data, identify the functional patterns inferred from sampled longitudinal child growth data and to study association.

The pattern from the FPCA gives a direct biological interpretation and offers a visual tool to assess the main directions in the functional data. The FPCA approach were shown to be providing a better estimate compared to other conventional methods to handle longitudinal data in biomedical applications [1, 2, 9, 10, 21, 35] and characterise trajectories in order to classify the pattern in child growth study [9] and various field of studies [12, 22, 23, 28, 33–35, 37–39].

In the present study, total variation was explained ≥90% for height and weight respectively. In literature review, application of FPC analysis are reported more than 80% of the total variation in multiple studies [9, 34, 38, 39]. In fact, we know that the growth outcome measurement obtained every month for 3 years exhibits 40% of the children had poor growth in the slum area, Vellore. Similar finding of high proportion of children had poor growth in their young age were observed in urban slum in India [9, 19, 40–44]. Our findings are similar to the existing literature in identifying the subgroup pattern such as “large, catch up, stunting, faltering and average” for the growth outcomes of height, weight, and head circumference. However, existing literature has not reported the percentage of individuals belonging to these subgroups of growth pattern [9]. One of the limitation in the application of this approach to sparse data is the need for numerical computation methods. 

There are many factors associated with the poor growth of children’s such as inadequate breastfeeding, parent’s socio economic status, family environment, and poverty. In this study, relating the more time points with a functional model will provide a complete and accurate figure as to how the study factors individually affect growth functions. The factors such as gender, socio economic status, breast feeding, education, and gravida are important and shown to be associated with growth outcomes [40–44]. There are many steps will be followed to make the information from the curves, but fewer options only available for developing inferences concerning predictor–outcome relationships, in hypothesis-testing of medical studies. The functional regression coefficient will be gained through figure from the modelling of functional regression, especially in the functional outcome and covariates. This model is still needed to develop more and we can consider this as a limitation. For functional inference, the coefficient plots are produced to each level of factors to understand the category nature over time in terms of function but the theoretical foundations for this area have not yet been developed.

## Conclusion

Longitudinal studies plays a vital role to recognize the growth pattern of children. In this study, we have used FPCA methodology for the child growth data and the growth FPC was obtained with biological interpretation. FPCA provides a useful methodology for the purpose of analysing growth outcome trends because it deals with subjects’ dynamics of growth and not with specific values at given times. Using this technique, we identified growth trajectories in order to discriminate the children who have normal growth and who have poor growth. Based on the first 3 years of child growth trajectories, we have found that majority of the children are comes under the greater risk of poor growth pattern among urban slum dwellers in Vellore, India. Occurrence of poor growth in young children will continue in the rest of the following months and will affect the child growth in later development stages. Our study using FPCA for identifying the poor growth pattern supports findings from previous studies [45–48] which helps make the policy decision in the government level to prevent the poor growth in future. Functional outcome linear regression model also useful to assess the factors association with long-term growth functions. The proposed regression in the research work addresses an extensive class of problems due to high-dimensional longitudinal data.

## Supplementary Information

**Additional file 1.**

**Additional file 2.**

**Additional file 3.**

**Additional file 4.**

**Additional file 5.**

**Additional file 6.**

**Additional file 7.**

**Additional file 8.**

**Additional file 9.**

**Additional file 10.**

## Data Availability

The datasets used and/or analysed during the current study are not publicly available due to institutional policy. Data are however available from the authors upon reasonable request and with permission of institution.

## Abbreviations

FDA: Functional Data Analysis
FPC: Functional Principal Component
FPCA: Functional Principal Component Analysis
FLM: Functional Linear Model
GCV: Generalized cross-validation
ICU: Intensive Care Unit
IRB: Institutional Review Board
LOCF: Last Observation Carry Forward
ANOVA: Analysis of Variance
SD: Standard Deviation
