# Supplementary figures and images for: Functional principal component analysis for identifying the child growth pattern using longitudinal birth cohort data
Source: BMC Med Res Methodol. 2022 Mar 21;22:76. doi: 10.1186/s12874-022-01566-0 (PMC8935724; doi:10.1186/s12874-022-01566-0)

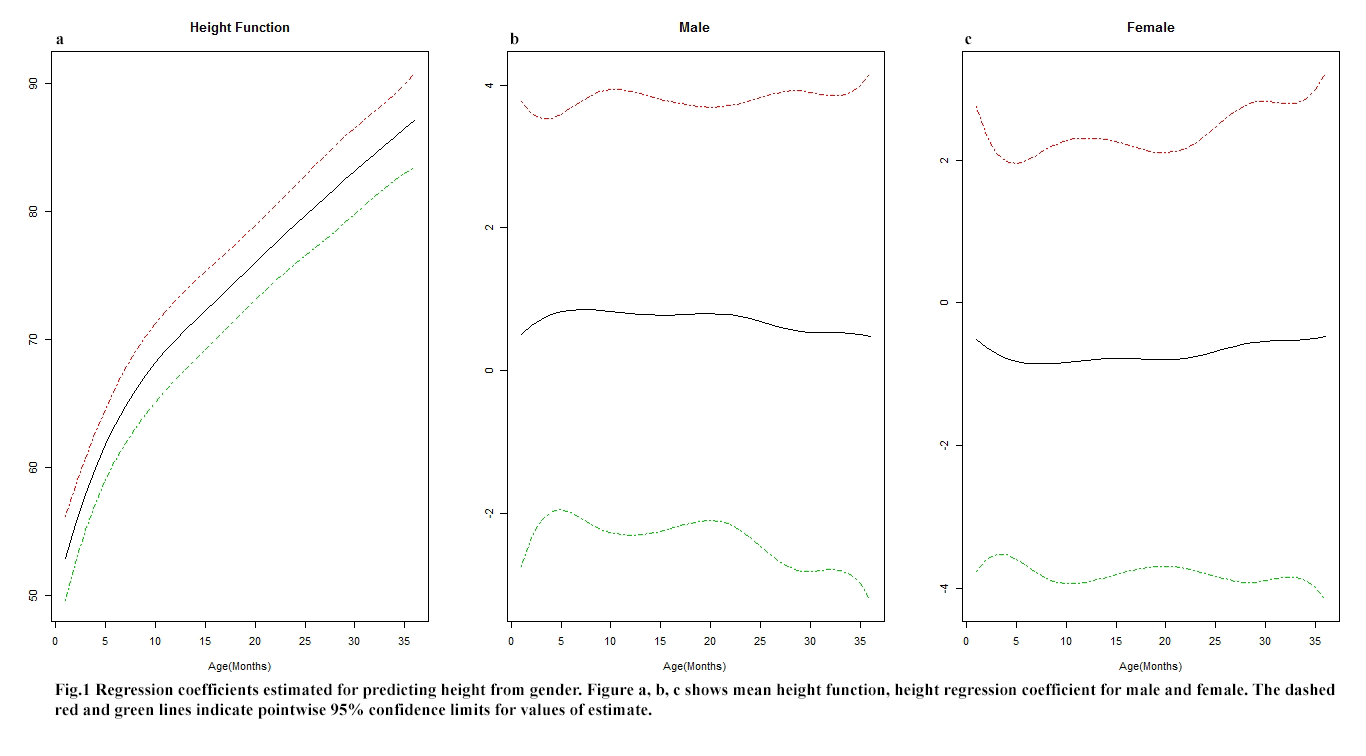

Supplement: Supplementary file 1 — Additional file 1. [file 12874_2022_1566_MOESM1_ESM.tif]

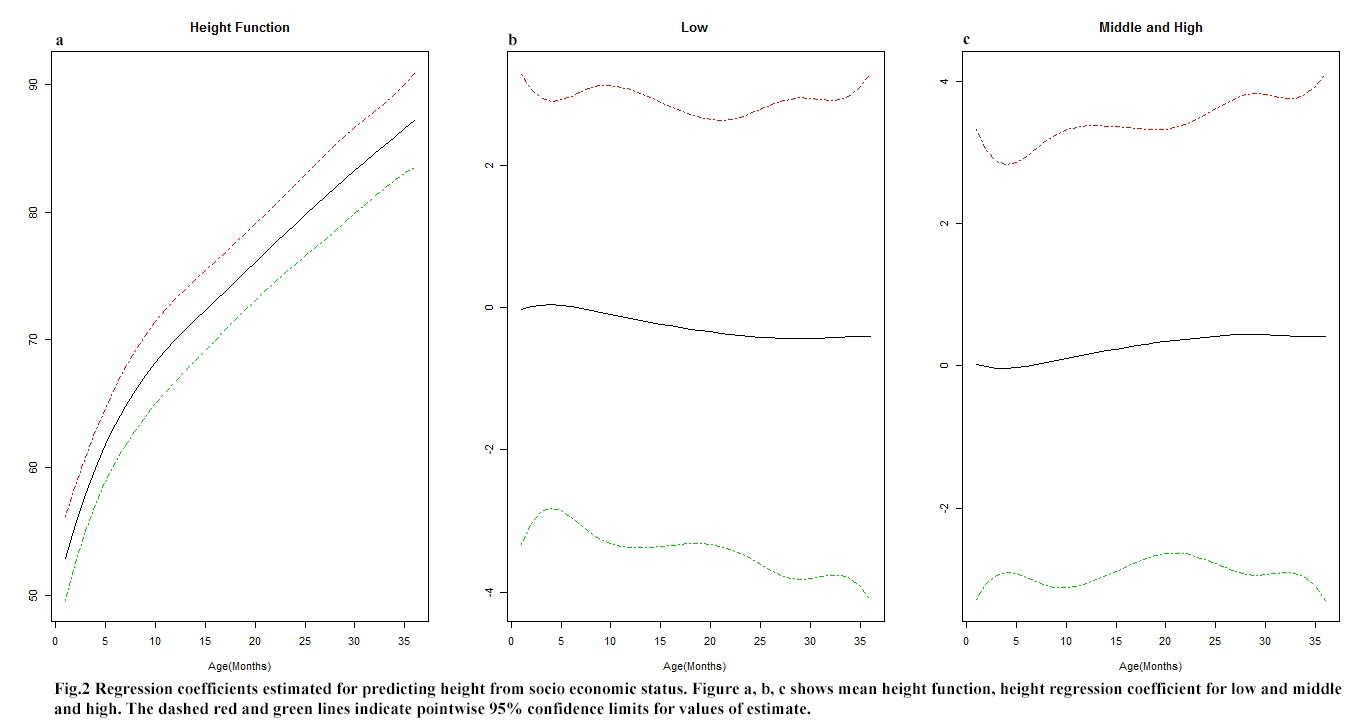

Supplement: Supplementary file 2 — Additional file 2. [file 12874_2022_1566_MOESM2_ESM.tif]

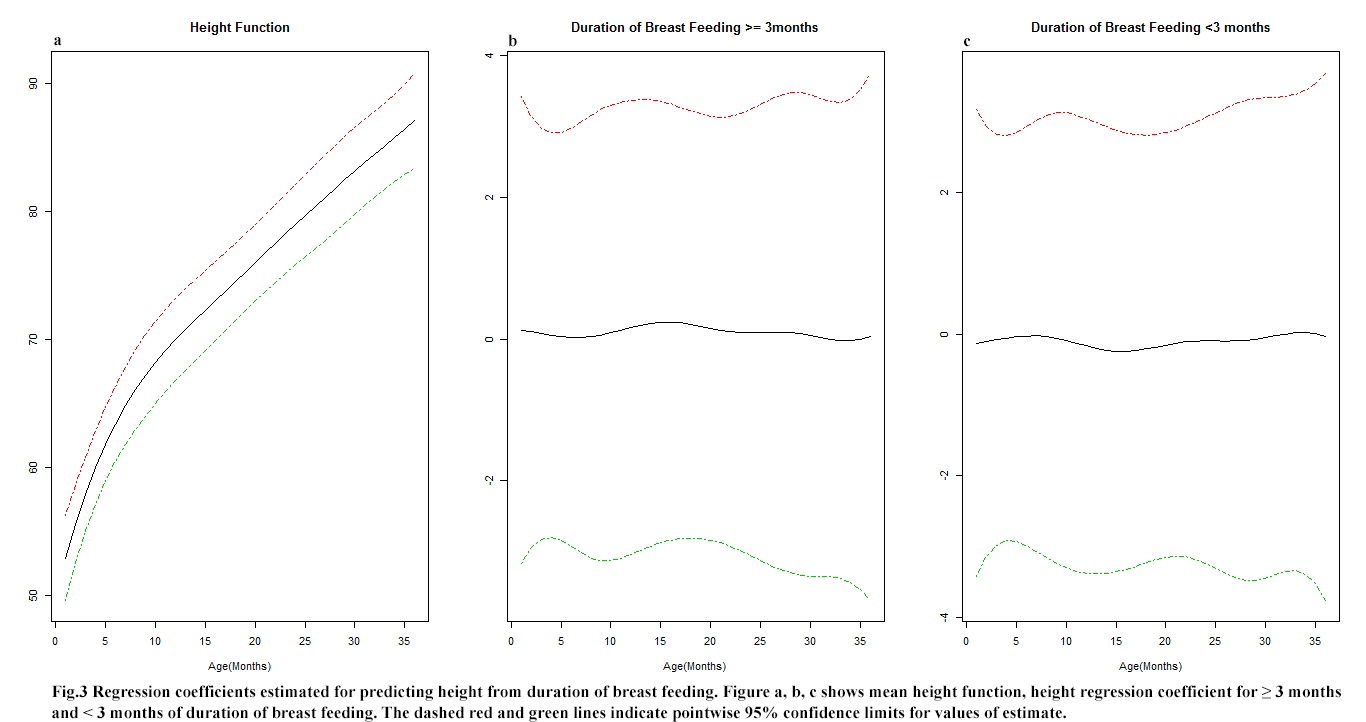

Supplement: Supplementary file 3 — Additional file 3. [file 12874_2022_1566_MOESM3_ESM.tif]

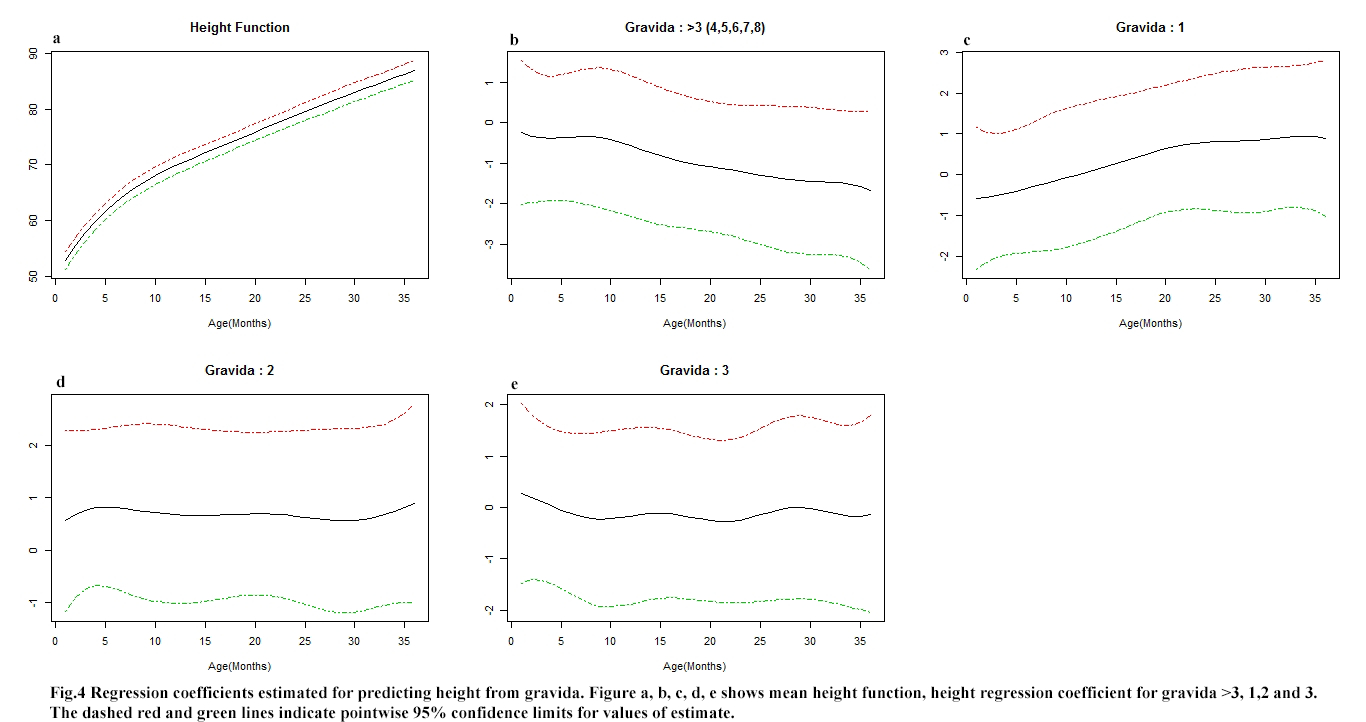

Supplement: Supplementary file 4 — Additional file 4. [file 12874_2022_1566_MOESM4_ESM.tif]

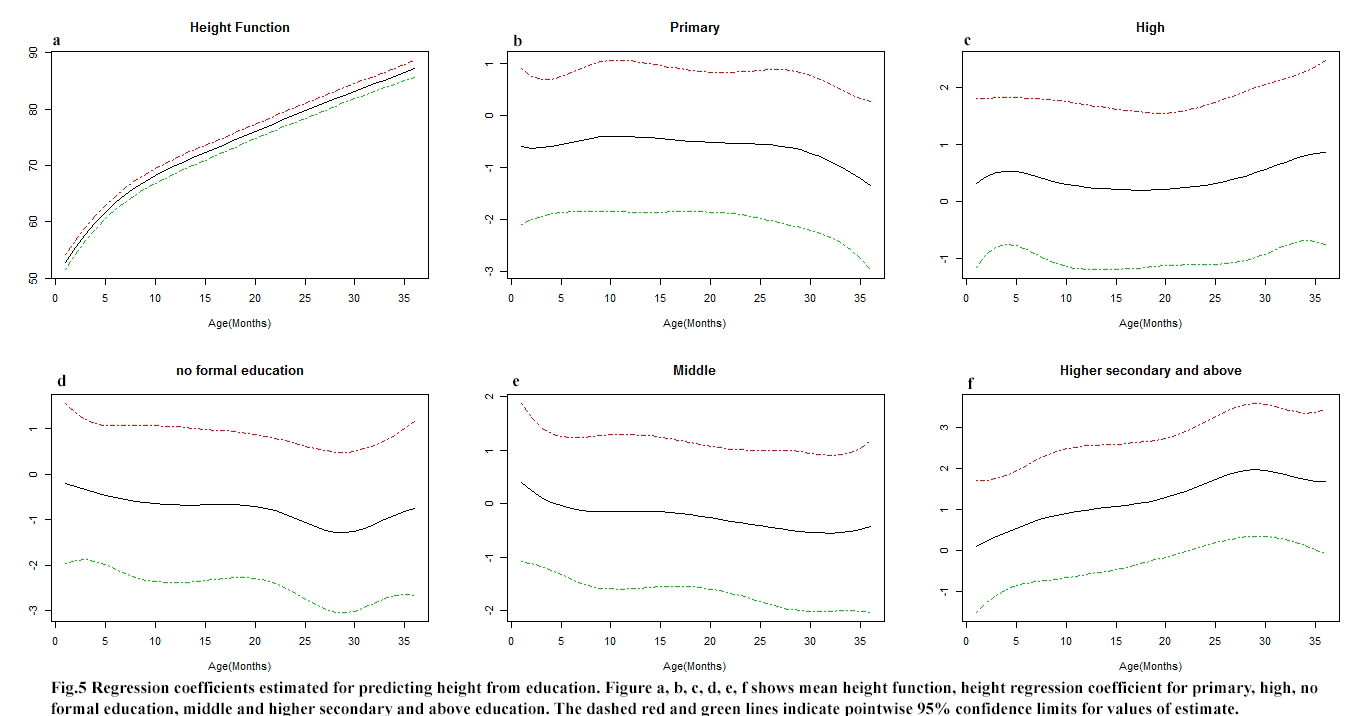

Supplement: Supplementary file 5 — Additional file 5. [file 12874_2022_1566_MOESM5_ESM.tif]

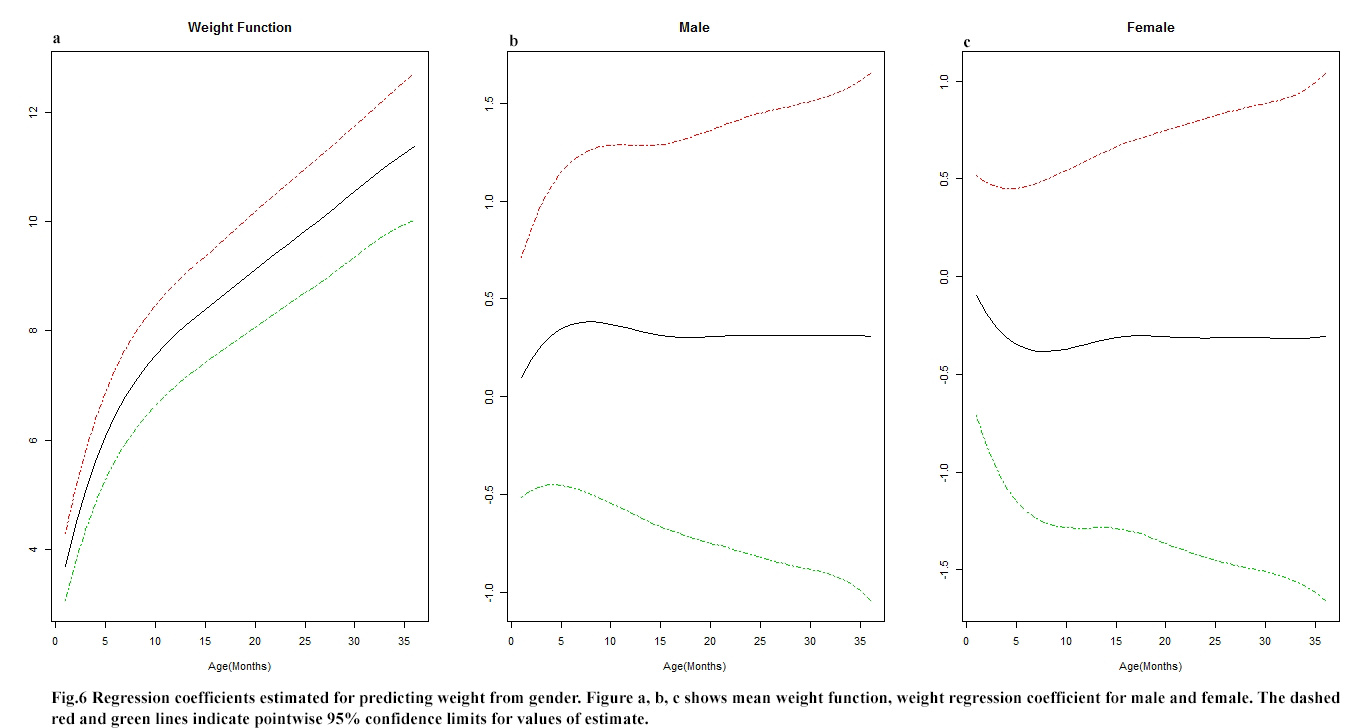

Supplement: Supplementary file 6 — Additional file 6. [file 12874_2022_1566_MOESM6_ESM.tif]

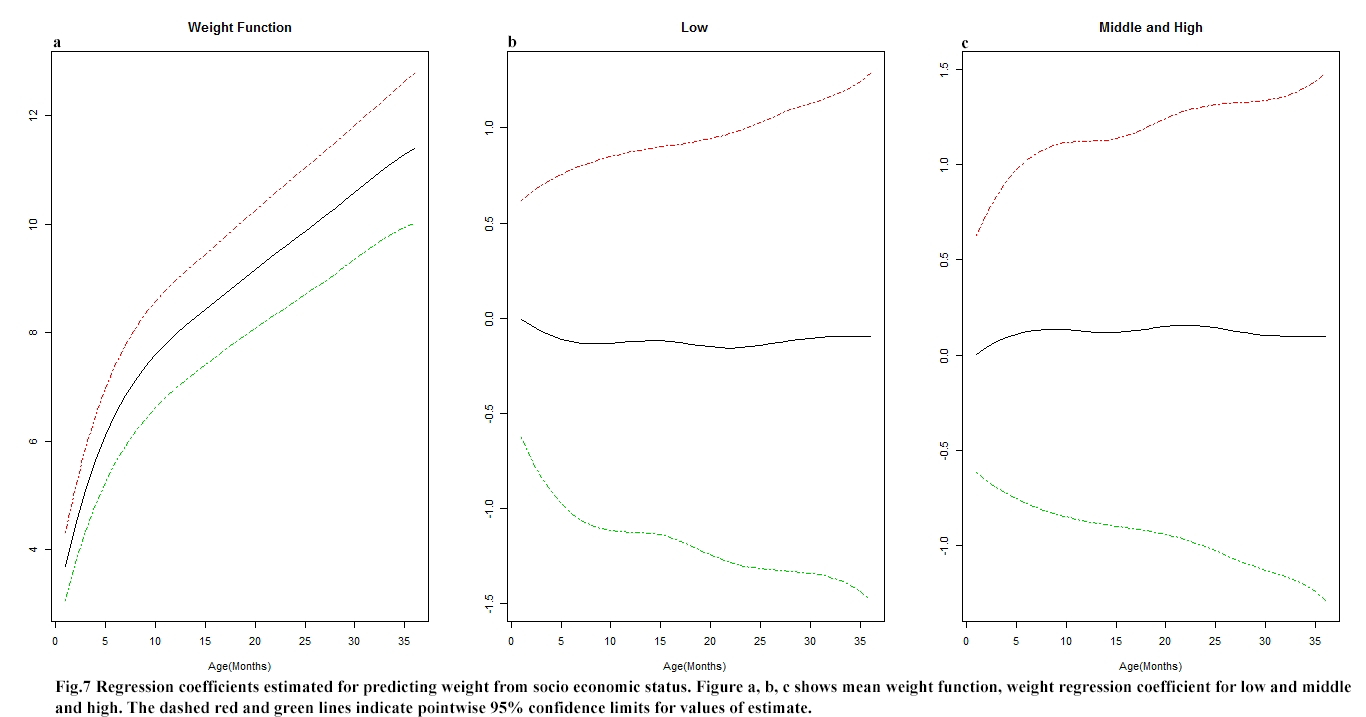

Supplement: Supplementary file 7 — Additional file 7. [file 12874_2022_1566_MOESM7_ESM.tif]

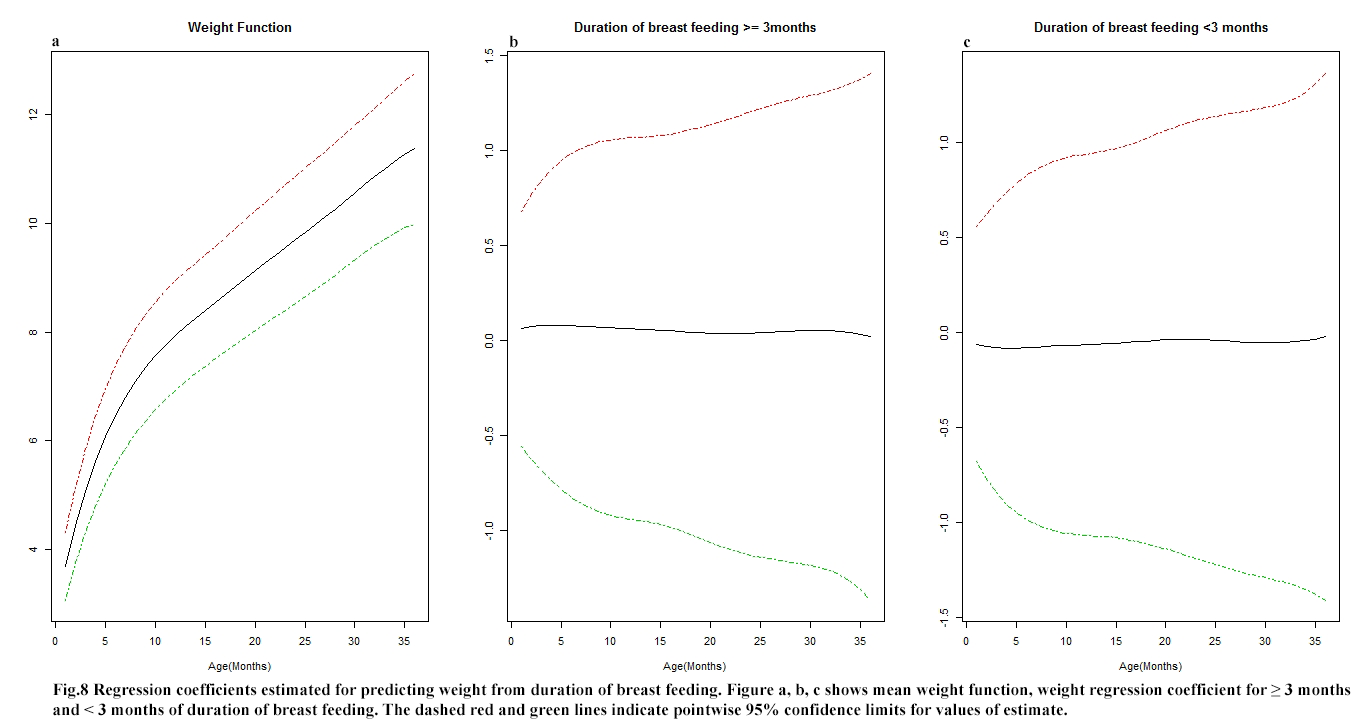

Supplement: Supplementary file 8 — Additional file 8. [file 12874_2022_1566_MOESM8_ESM.tif]

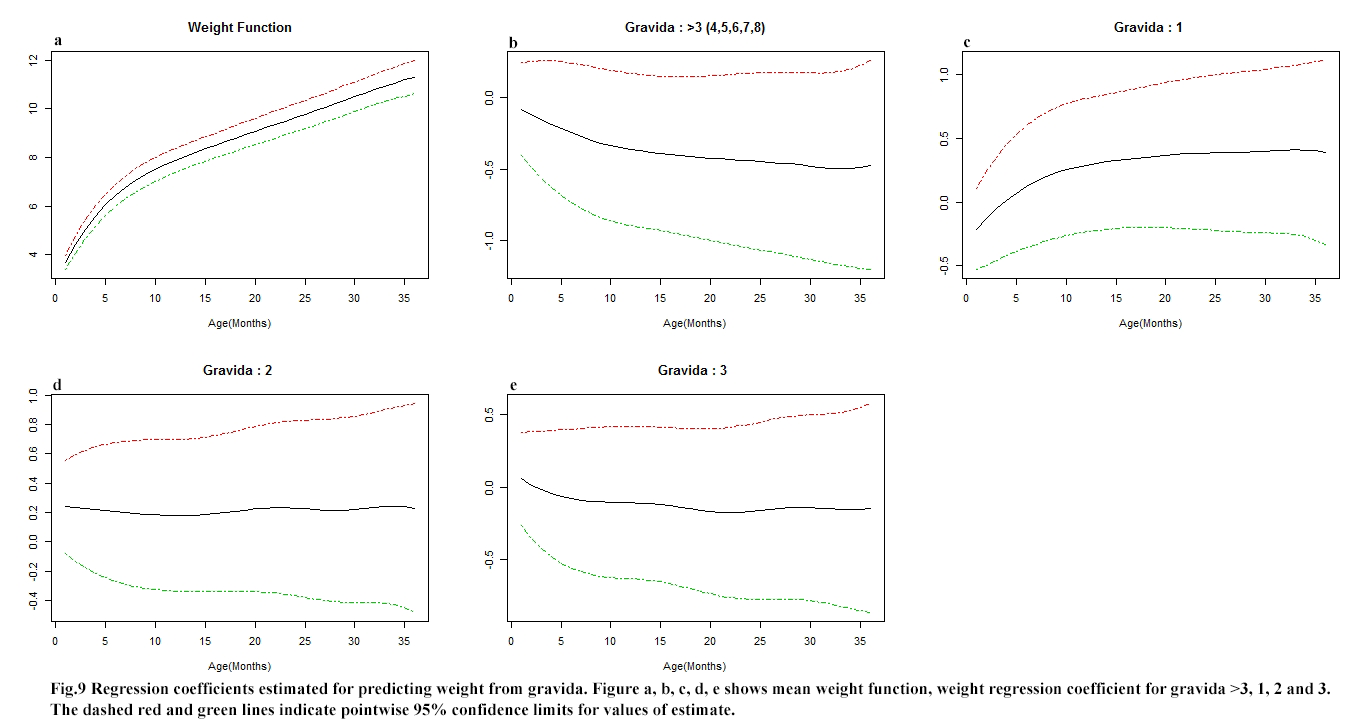

Supplement: Supplementary file 9 — Additional file 9. [file 12874_2022_1566_MOESM9_ESM.tif]

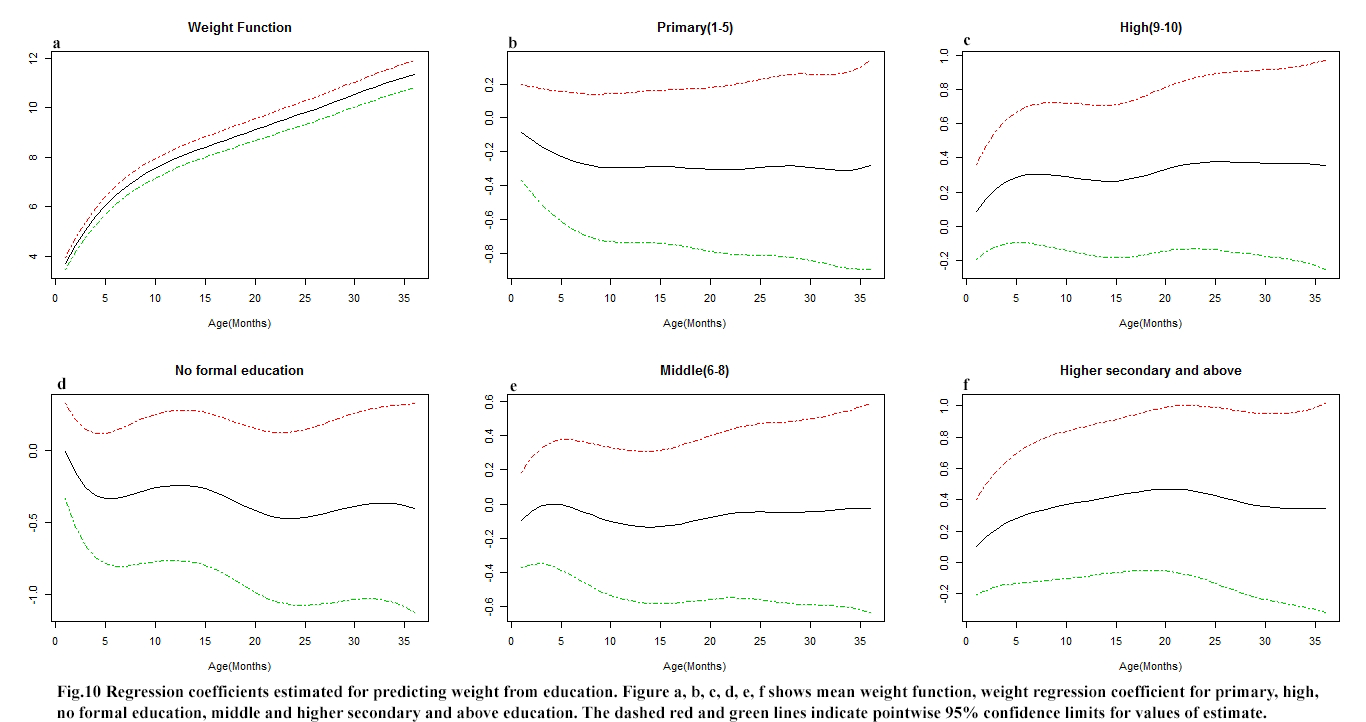

Supplement: Supplementary file 10 — Additional file 10. [file 12874_2022_1566_MOESM10_ESM.tif]
